# Psychiatrists’ Perceptions of the Role of Journalists in Suicide Reporting and Prejudices about Mental Illnesses in Portugal

**DOI:** 10.1007/s10900-024-01343-8

**Published:** 2024-02-27

**Authors:** Eudora Ribeiro, António Granado

**Affiliations:** ICNOVA – Instituto de Comunicação da NOVA, Universidade Nova de Lisboa, Lisboa, Portugal

**Keywords:** Suicide, Prevention, Mental health, Media, Journalism

## Abstract

International studies and the World Health Organization call for collaboration between media and mental health professionals to reduce the risk of imitative suicidal behaviour after suicide reporting – known as the Werther effect – and encourage individuals at risk to seek help. This study explores Portuguese psychiatrists’ perceptions of the practices of journalists, their interaction with those professionals, and their perspectives on the national suicide coverage through an anonymous online questionnaire and ten semi-structured interviews. The questionnaire received 128 responses. Only about 24% of the Portuguese psychiatrists characterized their relationship with journalists as cooperative, and most of them considered suicide reporting to be sensationalist and irresponsible. More than 80% of the participants expressed the view that journalists do not know the guidelines for responsible suicide reporting, but more than 95% considered that they can contribute to suicide prevention. These findings suggest that there is a long way to go to build a constructive partnership for suicide prevention between psychiatrists and journalists in Portugal, focused on improving the quality of suicide reporting. We hope this study may inspire similar studies in other countries, since suicide is an international public health problem and collaboration between media and mental health professionals can help to prevent it on a global scale.

## Introduction

Suicide is not merely attributed to a mental illness experienced by the individual but also to the influence of the social environment [1]. Indeed, individuals live within families, schools, professional settings, neighbourhoods, and countries, which introduce risks or protections for population health [2]. Furthermore, socioeconomic factors affect both physical and mental health, through mechanisms that include employment, exposure to crime or pollution, access to physical activity, and availability of healthy food [3].

Suicide is the result of social and structural factors, rather than individual ones, in two major domains: social integration – the social bonds of the individual with others and with society – and moral regulation – the effects of societal norms on individuals [4]. Contemporary reports have extended Durkheim’s insights, notably that individuals’ membership in a specific group determines their vulnerability to suicide based on the collective’s integrative and regulative characteristics, linking them to individual feelings or beliefs about who they are and what they are supposed to do [5].

Durkheim’s conceptualization can also help to frame increases in suicides following media reporting of deaths by suicide, especially of celebrities. Decades of research have confirmed that exposure to media reports of suicides is associated with an increased risk of suicide [5], known as the Werther effect [6], especially if the coverage is extensive, prominent, sensationalist, and explicitly describes the method used [7, 8].

Multiple studies from various countries have reported suicide rises following reporting of celebrity suicides [9], such as those of Marilyn Monroe, Robert Enke, Robin Williams, Kate Spade, and Anthony Bourdain [10–17]. Vulnerable individuals are the most at risk of being influenced to engage in imitative behaviour following media reports of suicide [7, 8].

However, responsible reporting on suicide can contribute to suicide prevention, a phenomenon known as the Papageno effect [18], since it may serve to educate the public about suicide and its prevention, encourage individuals at risk to seek help, and promote more open dialogue about suicide [7, 8]. Responsible reporting on suicide can be described as the avoidance of harmful elements that may trigger suicidal contagion and the inclusion of helpful content that may prevent suicides within stories [19]. In this sense, the role of media professionals is of the upmost importance [18].

Several countries and the WHO itself have developed media guidelines to facilitate responsible suicide reporting. The WHO first released recommendations to media professionals in 2000, which were updated in 2008 and 2017. However, in many cases, professional conventions and routines do not conform to the guidelines for responsible suicide coverage.

Some studies have already asked journalists for their opinions on suicide reporting and these recommendations. Most German journalists spoke of a role conceptualized based on maximum journalistic freedom, and some felt the media guidelines restricted that freedom [20]. Canadian media professionals also considered that suicide reporting guidelines conflicted with some of the core values of media culture, and they questioned the media’s role and responsibility in suicide reporting, suicide prevention, and mental health dialogue, perceiving the guidelines as a form of censorship [21].

American journalists were equally concerned about the potential pitfalls of restrained suicide coverage, although they were not insensitive to worries about potential contagion effects [22]. Irish journalists, for their part, were not explicitly concerned with avoiding harm but welcomed guidelines and continuing guidance from organizations to address the lacuna in mental illness reporting [23]. Portuguese journalists wanted to promote public discussion of suicide and cooperate with mental health specialists [24].

Several international studies highlight the importance of and need for collaboration between media and mental health professionals to reduce the risk of imitative suicidal behaviour and encourage seeking help [9, 15, 25–34]. Awareness raising among both professional groups regarding the significance of this cooperation could start in pre-professional education [24, 35] or early carrier [36]. For instance, by working in collaboration, Australian mental health professionals and journalism educators have contributed to a curriculum resource with the purpose of improving journalists’ skills in reporting on mental illness and suicide [37]. The WHO [7, 8] advises media professionals to seek the advice of experts in mental health when preparing stories about suicide. Mental health specialists are also encouraged to help the media in suicide reporting [35, 38, 39].

There are historically high suicide numbers in some regions of Portugal [40]. However, Portuguese psychiatrists have never been surveyed as a class on suicide reporting. To fill this gap, this study intends to explore their perceptions of the practices of Portuguese journalists, their interaction with these professionals, their perspectives on the national suicide coverage, and what can be done to make it more responsible. It is our intention to contribute to the debate around the improvement of suicide reporting with a preventive purpose and stimulate reflection about the role of mental health specialists, such as psychiatrists, in this regard: Could they play a more active role? What do they think about that? Do they feel prepared? What can be done towards that end?

## Methods

### Study Design

We decided to choose, from the start, a mixed-method design, with two complementary methods: first, an online questionnaire; second, semi-structured interviews. Our aim with the online survey was to collect opinions from the greatest possible number of Portuguese psychiatrists and examine them as a professional class. The interviews were intended to further explore some points of the online questionnaire, allowing for and encouraging the sharing of more personal views and experiences.

### Questionnaire

An online questionnaire was prepared for distribution to Portuguese psychiatrists, comprising mainly closed-ended and multiple-choice questions, all mandatory. Many of the questions were based on the WHO guidelines for media professionals for responsible suicide reporting, which are the basis for the national recommendations. For instance, we asked psychiatrists to evaluate suicide reporting in Portugal against some of the main WHO guidelines, to judge the performance of Portuguese journalists and its potential for suicide prevention. Most of the questions (11) were designed as a yes/no question, some (7) used five-point Likert scale statements (Totally agree, Agree, Neither agree nor disagree, Disagree, Totally disagree), and others were multiple-choice (4). There were no questions about psychiatrists’ experience with suicidal patients.

A partnership was established with the Portuguese Medical Association, specifically with the College of the Specialty of Psychiatry, which discussed and approved the online survey in a plenary meeting and subsequently sent the questionnaire by email to the 1,522 registered psychiatrists. The questionnaire was also distributed to the national psychiatry interns by the Portuguese Association of Psychiatry Interns (APIP). Responses were accepted between 6 September and 31 October 2021. To avoid double inclusion, a participant’s email was automatically excluded once that participant completed the online questionnaire.

### Interviews

To complement the questionnaire data, ten semi-structured interviews were carried out, in person or by video call, between 4 January and 2 May 2022. Four of the interviewees expressed their availability to be interviewed by email, after answering the online questionnaire. We created an email address to receive messages from volunteers for interviews, which was placed in a disclaimer at the end of the online questionnaire.

The other six doctors were selected using a purposeful snowball sampling, considering their professional experience and knowledge of the subject, whether through research, publications, books, or community involvement in suicide prevention initiatives. All the doctors were in favour of no anonymity in their answers, to counter the taboo around suicide and stimulate the debate around the topic. All the participants provided verbal informed consent, which was witnessed and formally recorded.

The semi-structured interview guide was based on the online survey questions, but they were transformed into open questions to allow the interviewees to freely express their opinions, perspectives, and personal experience. The quotes presented throughout the text below are meant to complement the results of the questionnaire.

The interviews were conducted by the first author. Most lasted between 30 min and 1 h. All interviews were audio-recorded. The transcripts were checked twice for accuracy by the first author and coded for recurring concepts or ideas in the respondents’ answers.

## Results

Among the psychiatrists interviewed, five were women and five were men, with 5 to 38 years of professional experience. The number at the end of every quotation in this article represents the years of professional experience of each doctor. The interviewees came from nearly every part of the country (except the islands). There were 128 responses to the questionnaire from psychiatrists across the country (including the islands). Almost 59% of the respondents were women and the remaining 41% were men. The psychiatrists’ ages ranged from 26 to 81 years, with an average age of 47 years. The respondents’ average professional work experience was 20 years.

Nearly 62% of the respondents to the online survey characterized their relationship with Portuguese journalists as neutral, about 24% as cooperative, and 14% as conflictual.

“The relationship is a bit utilitarian, opportunistic, and personal” (30), one interviewed doctor said. Another psychiatrist stated that the interaction between psychiatrists and journalists “is very weak, very tenuous” and “there is somewhat of a divorce between psychiatrists and journalists” (38).

We found a significant correlation (*r* = 0.22, 95% CI [0.045, 0.38] *p* = 0.014) between the respondents’ age and professional experience and their perception of the relationship between psychiatrists and journalists: as psychiatrists get older and more experienced, they show a greater tendency to see that relationship as cooperative.

Several psychiatrists talked of communication barriers or difficulties, in particular an “addiction” of psychiatrists “to communicating in a certain way”, without adjusting their speech in order to be better understood, as one specialist said (11). One doctor said that psychiatrists are generally available to talk to journalists, while admitting doubts as to whether they are “able to convey a clear message that is useful to people” (6). Another one alleged that during their education and training psychiatrists are not prepared to contribute to health literacy, they are only “formatted to see patients” (6).

The news media were also criticized: “Some things are not explained correctly, or journalists transmit only the most striking part rather than everything that is important to define a certain concept or disease” (11). Another psychiatrist considered that “the word suicide is very contaminated in the national media”, as exemplified by its use by political commentators “to describe somewhat unfortunate political moves” (30).

Despite admitting the existence of prejudices between journalists and psychiatrists, several interviewed psychiatrists noted that journalists and psychiatrists need each other. One doctor even considered that “psychiatrists have a duty to work with journalists”, to demystify their profession. “Patients think that we are bogeymen. If we work with journalists, we also demystify the stigma of mental illness” (5), she added.

All the interviewed doctors agreed that there are many prejudices and stereotypes about mental health issues, both in the population and among psychiatrists themselves. “Sometimes it [suicide] is taboo because we associate it a lot with a weakness, that being mentally ill is being weak in the head or not being reliable” (11), one specialist said. Another psychiatrist noted that thinking that a psychiatric illness can lead to suicide is quite scary and that is why there are so many prejudices, which he explained as “easy answers to doubts and fears” (18).

About two thirds of the Portuguese psychiatrists who answered the online survey held the view that suicides should not be in the news, while 33.6% answered that they should, and the questionnaire then included items intended to explore the reasons why. No significant correlations were found between the answers and the age, sex, and number of years of professional experience of the respondents.

Furthermore, the majority (58.6%) of respondents considered that the risks of potential copycat effects should have more weight in the decision on whether to report on suicides, while 41.4% considered that the population’s awareness of suicide and mental health should matter the most. The underlying problem is well known among Portuguese psychiatrists: the risk of copycat behaviour among vulnerable individuals after suicide reporting with certain features.

However, many of the interviewed doctors were against not talking about suicide in the news media, notably because of the potential (preventive) Papageno effect of media reports that follow certain guidelines for responsible suicide reporting. One psychiatrist said that “suicide must be talked about, because not talking is a conspiracy of silence” (5). Another argued that “suicide can be reported, but the available psychiatric services should always be mentioned, and the medical aspect must always be highlighted, because there is a brain disease behind it” (10).

**Fig. 1:**
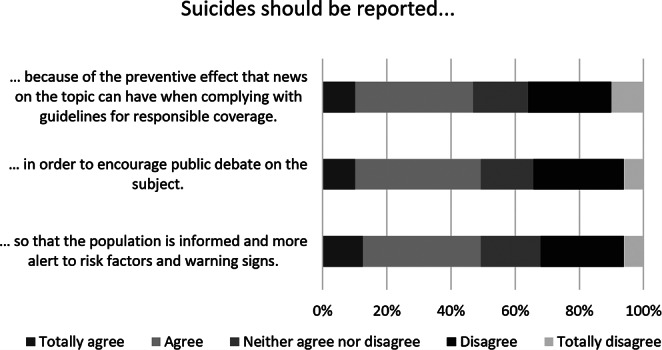
Opinions of 128 psychiatrists on reasons to report suicides

**Fig. 2:**
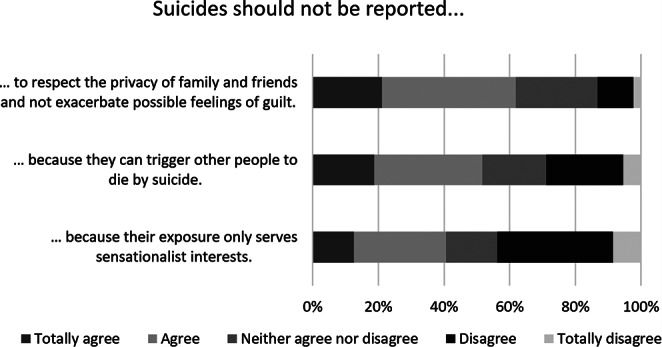
Opinions of 128 psychiatrists on reasons not to report suicides

In the online survey, psychiatrists were also asked to indicate the reasons why they believed suicides should or should not be reported using six five-point Likert scale statements. Fig. 1 shows that most of the surveyed doctors claimed to support suicide reporting for the purpose of informing the population and making it more alert to risk factors and warning sings (49.2%) and to encourage public discussion of the topic (49.3%). Almost 47% believed that reporting on suicide can have a preventive effect. “It’s important to inform the population, so suicide must be integrated in the context of mental health, explaining that it is possible to help people in crisis” (11), one psychiatrist said. About 52% of the Portuguese doctors were not in favour of reporting on suicides because of the potential for copycat behaviour (Fig. 2).

**Fig. 3:**
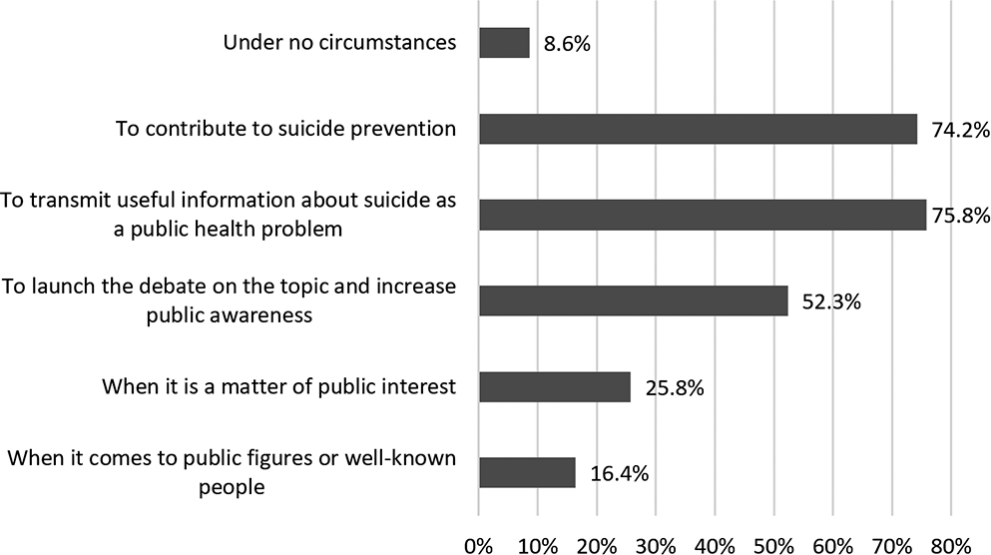
Opinions of 128 psychiatrists as to the circumstances under which suicides should be reported

Regarding the circumstances under which suicide should be reported, almost 76% of respondents believed that suicide should be reported to transmit useful information about it as a public health problem; 74.2% thought that it should be reported to contribute to its prevention; and 52.3% considered that it should be reported to launch a debate about the subject and increase public awareness (see Fig. 3).

One interviewed psychiatrist noted that “talking about suicide for informational, educational, and pedagogical purposes is a mission of the media” (30). Another doctor stated that “news about suicide, compliant with the rules, including warning signs and risk factors, should be reported to increase the mental health literacy of populations” (33).

Although only 16.4% of the online survey respondents answered that suicides should be reported when they involve public figures or well-known people, most of the interviewees admitted that someone’s suicide is news if that person has an important role in society, “but it should never be glorified, nor should there be a sensationalist exploitation of the events” (38), and those news must “offer alternatives to suicide, such as the treatment of mental illnesses, and social support resources, such as helplines”, so that people at risk can find help (6).

More than 80% of the online survey participants considered that Portuguese journalists do not know the WHO guidelines for responsible suicide reporting. Several interviewed psychiatrists were against censorship and prohibitions, but many defended including WHO recommendations in journalism education and training. One doctor argued that “journalists must be educated about the rules, and the importance of talking about these issues must be explained to them, because they have a fundamental role in health literacy” (6).

Most of the respondents to the questionnaire described suicide reporting in Portugal as sensationalist (67.2%), irresponsible (68%), not complying with the best recommendations (75%), nor assuming a preventive role (85.2%), and underreporting the connection between suicide and mental health (69.5%). “It’s too bad, I haven’t seen anything particularly well done about suicide. It’s all very sensationalist, simplistic, or it’s not talked about at all” (5), one interviewed doctor said.

Almost 54% of the doctors surveyed online considered suicide a taboo in Portuguese journalism, which for 36% of them stems from it being a taboo in society and for 18% of them from the associated stigma. All the interviewees considered suicide a taboo. One psychiatrist said that “it’s very much a taboo, very little is said, even among doctors” (18), and another shared that “when a doctor dies by suicide, there is a deafening silence” and “nobody has the courage to talk about it” (5).

Most interviewed psychiatrists underlined that the media do not talk enough about suicide, mostly for fear of doing it wrong due to not knowing what is at stake or how to do it without the risk of imitative behaviour. “Either the press doesn’t refer to anything at all, which is a failure because it increases the risk of stigma around psychiatric disease, or it does it in an unfortunate way” (10).

Nevertheless, most of the interviewees considered that suicide reporting in Portugal has been evolving positively in the last decades, although they noted that “there’s some excitement that should be avoided” when famous people are involved (18).

The online survey has also shown that most of the Portuguese psychiatrists (62.5%) believe that suicide reporting can contribute to reducing the stigma surrounding suicide, and more than 95% answered that the media can promote dialogue and awareness about public health problems, such as suicide, and that journalists can contribute to suicide prevention by promoting public education.

One interviewed doctor stated that “journalists can indeed be a vehicle for reducing stigma because they are able to bridge the gap with the public through an adequate language to pass on information” (10). Another psychiatrist argued that “a lot of the stigma around mental health and suicide comes from insufficient information and from cliches and prejudices that people have internalized and that good journalism can help to combat” (25). In this sense, the same doctor underlined that the collaboration between journalists and psychiatrists can be very helpful in the prevention of mental illness and suicide.

Portuguese psychiatrists also seem to understand the conflict between the journalistic practice and the recommendations for responsible suicide reporting. “Journalists are always looking for details. This is almost understandable and even to be expected” (38), one specialist said. In this sense, one doctor stated that there is a need to “find a balance between delivering the news in the way journalists learned to do it and complying with suicide reporting guidelines” (6).

## Discussion

To our knowledge, this is the first study with psychiatrists on suicide reporting and their interaction with journalists. The results suggest that there is a long way to go to build a constructive partnership for suicide prevention between Portuguese psychiatrists and journalists, focused on improving the quality of suicide reporting in Portugal. The fact that only about 24% of the surveyed psychiatrists characterized their relationship with journalists as cooperative is particularly revealing. Moreover, the fact that almost 68% of the surveyed doctors described suicide reporting as sensationalist and irresponsible is a worrying finding. However, this perspective is in line with the views expressed by most Portuguese journalists [24] and with the reality perceived by Hong Kong journalists [41], New Zealand media professionals [42], and Canadian professionals [21]. The reason for it is simple: in increasingly competitive news markets, media organizations may not act responsibly but rather choose to let sensationalist news coverage speak on their behalf [43].

Therefore, it is urgent to train psychiatrists in the proper way of interacting with the media so as to make media professionals aware of the serious impact of negative suicide reporting on many vulnerable individuals [35]. Short sessions of media training can be effective for improving the ability of psychiatrists to guide journalists toward a more responsible media coverage of suicide [44]. Several of the Portuguese psychiatrists who were interviewed also suggested various ways of strengthening the interaction between journalists and psychiatrists, like annual colloquiums and/or regular training actions. Periodic joint workshops run by mental health professionals and media personnel are also recommended to support responsible reporting [33].

Indeed, collaboration between mental health and media professionals is essential to redress some knowledge gaps, particularly regarding how suicide stories are produced, what information they contain, and how suicide is framed [30]. This is especially important to the extent that most journalists state they do not know the existing guidelines for responsible suicide reporting and admit there is a lack of training on suicide reporting guidelines during pre-professional education. This is the case among Portuguese [24], British [45], and American journalists [22].

Furthermore, public perception of the field of psychiatry, and of mental illness itself, is generally not positive, and the stigma of mental disorders seems to be coupled with an attitude of suspicion from the public towards psychiatric practitioners [39]. Several interviewed doctors acknowledged that their collaboration with journalists could help demystify mental illness, as well as their own professional activity, as they are seen by many as a kind of “bogeymen”.

Psychiatrists should be encouraged to interact with the media to offer scientific information about mental illnesses. Likewise, methods and principles of interaction with the media should be part of psychiatrists’ training curriculum, since statements to the media can be “a double-edged sword” [35] and reluctance by physicians to enter the media battle is not new [26].

As one Portuguese psychiatrist put it: “It’s possible to have good relations between psychiatrists and journalists” (30). Media professionals and suicide experts must work together to balance newsworthiness against the risk of copycat behaviour [31]. Journalists certainly face the challenge of bringing greater profile to mental health issues, notably suicide, in a way that both respects their professional duties and considers the potential harmful effect of that coverage on vulnerable individuals [46].

Few public health problems have been solved with silence. On the contrary, the media can be an ally in promoting dialogue and raising awareness of public health problems such as suicide [47]. In fact, 83.6% of the Portuguese doctors surveyed agreed (36.7%) or totally agreed (46.9%) that silence does not help to solve public health problems.

This study has also shown that Portuguese psychiatrists seem to understand the conflict between the journalistic practice and the recommendations for responsible suicide reporting. This view is shared by psychiatrists in other countries, who recognize that recommendations on how to report suicide often run counter to the natural journalistic approach or instinct to report a story [48]. Psychiatrists even understand the media’s need to report and sensationalize news to increase their reach but point out that this reporting has an enormous impact on the mental health of vulnerable individuals [35].

## Limitations

The main limitation of our study is the response rate. There were 128 responses to the online questionnaire. From the universe of 1,522 psychiatrists registered with the Portuguese Medical Association, only 92 doctors (6%) answered. The remaining 36 responses were from psychiatry interns (about 11% of the total number of trainees in September and October 2021) [49].

Despite the low response rate, as far as we know, this is the first study with psychiatrists on suicide reporting and their interaction with journalists, and we believe that we have gathered enough responses to address the topic and share the results with the international community. The WHO and several international studies call for greater interaction between mental health and media professionals when it comes to reporting suicide. This should take the form of a direct contact with clinicians, who are considered the most fitted to talk about suicide, its prevention, and the associated mental illnesses.

Another limitation is the purposeful sampling regarding some of the interviewees. However, to ensure the anonymity of the online survey, it was not possible to recruit more doctors among those who had previously answered the questionnaire.

## Conclusions

Findings show that Portuguese psychiatrists, regardless of their age group and years of professional experience, are willing to cooperate more with journalists to increase public awareness of suicide and mental health literacy. This is a positive finding since they have criticized and negatively rated Portuguese suicide reporting and considered that journalists mostly do not know the guidelines for responsible suicide reporting. Another recent study has concluded that Portuguese journalists are also willing to cooperate more with mental health specialists, such as psychiatrists, to improve public awareness on suicide [24]. This seems to indicate that there is a basis for stronger collaboration between these two professional groups aimed at promoting more responsible suicide reporting in Portugal and contributing to suicide prevention.

This study seeks to stimulate the debate around the reporting of suicide and the role that psychiatrists can play in promoting mental health literacy and more responsible suicide reporting. Future research could investigate where this relationship between psychiatrists and journalists stands in other countries and whether, over time, there has been any change or improvement that could be associated with an evolving perception of mental health illness and suicide in the community itself.

## Data Availability

The data that support the findings of this study are available from the corresponding author on reasonable request.
